# The Coffee was Paid for Dearly: Shipwreck BZN4 and the Frigate*’t Hart*

**DOI:** 10.1007/s11457-025-09458-2

**Published:** 2025-12-24

**Authors:** L. Tran, J. Oosterbaan, A. D. Vos, V. Enthoven, S. van Daalen

**Affiliations:** 1 Leiden University, Leiden, The Netherlands; 2 Batavialand, Lelystad, The Netherlands; 3 Leiden, The Netherlands; 4 Van Daalen Dendrochronologie, Wijhe, The Netherlands

**Keywords:** Frigate, Casks, Marks, Merchantman, West Indiaman, Suriname

## Abstract

The Burgzand, located east of the Dutch island of Texel, has a rich maritime history. This area served as a part of the Texel roadstead, offering ships ample deep water and a sheltered anchorage. Despite its assumed safety, the Burgzand witnessed numerous shipwrecks, including the Burgzand Noord 4 (BZN4). Since 1999, archaeological expeditions have been conducted on the shipwreck, but this research has not yet led to an identification of the wreck. However, new research into the marks on casks found in the wreck, combining insights from other archaeological findings and textual sources, has revealed a very strong candidate for identification: the two-deck frigate*’t Hart*. Thus far, the West Indiaman BZN4, representative of the ships of the trans-Atlantic trade in sugar, coffee, and cocoa, is the only such ship discovered in Dutch waters. Identifying BZN4 as *’t Hart*, which is the main objective of this paper, would offer a unique opportunity to explore trade and shipping between the Dutch Republic and its South American colonies.

## Introduction

Identifying shipwrecks is notoriously difficult. To establish a wrecked ship’s name using historical sources, carefully analyzing archaeological finds can help refine the search, significantly increasing the likelihood of success. When successful, archaeological data from the shipwreck can be compared to data from textual sources, enabling a detailed understanding of the excavated wreck and its trade networks. Within the boundaries of Dutch waters, convincingly successful shipwreck identifications have remained relatively rare. In the few cases where identification has been possible, the wrecks have typically been linked to the Verenigde Oost-Indische Compagnie (VOC, Dutch East India Company), the West-Indische Compagnie (WIC, Dutch West India Company), or the Admiraliteitscolleges (navy).^1^ In such instances, the extensive archival records maintained by these institutions have played a crucial role in facilitating the identification process. In the case of merchant ships, the identification proves to be particularly challenging, given the scarcity of written records concerning these ships.

The waters of the Netherlands are littered with shipwrecks, which is not surprising considering its dominance in trade and shipping in the seventeenth and eighteenth centuries. This also applies to the roadstead of Texel, located in the Waddenzee directly east of the island. Estimates suggest that between 500 and 1000 ships were lost in this area from the 16th to the 18th century (Vos 2012, 47–55).

**Fig. 1:**
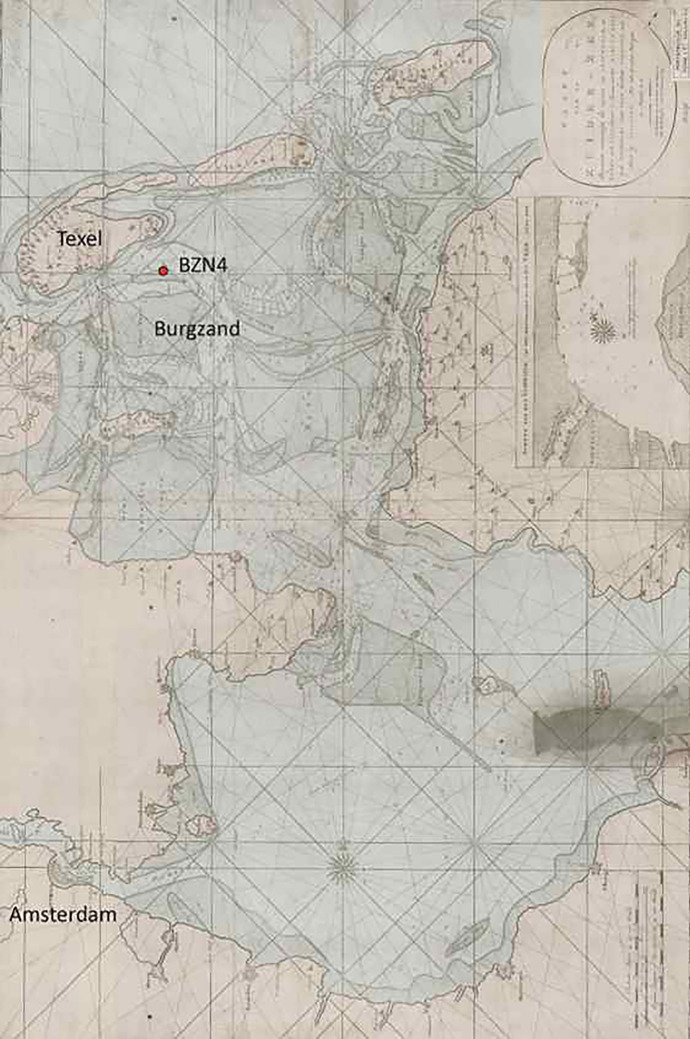
The location of the wreck BZN4 (N 53º 02.70’ O 4º 56.06’ (WGS 84); X = 124,628, Y = 562,107 (RD)) projected onto a map of the Zuiderzee (1780). *Source*: Noord-Hollands Archief, Collectie kaarten Rijkswaterstaat vóór no. 108, Kaart van de Zuiderzee behelzende voornamelijk het vaarwater van Amsterdam, na Texel ’en ’t Vlie, 1780

During this period, the deep waters east of Texel served as a frequent anchorage for large seafaring ships. Due to their deep draft, these ships could not cross the shallow Zuiderzee when fully loaded and anchored off Texel instead. Nevertheless, this location did not always provide a safe haven, as evidenced by the numerous shipwrecks, of which the vast majority remain unidentified. In this paper, we present strong evidence for the identity of one of the shipwrecks, known as Burgzand Noord 4 (BZN4) as the two-deck frigate*’t Hart* (Fig. 1).

Based on the archaeological findings, BZN4 has been identified as a merchantman on a voyage originating in South America. In its hold were numerous casks containing coffee beans, a typical product from the Dutch plantation colonies of the Wild Coast (Suriname, Berbice, and Essequebo), situated between the Orinoco and the Amazon. This ship remains the sole West Indiaman discovered in Dutch waters, making it important material evidence of the relationship between the Dutch Republic and its colonies in the eighteenth century. Dendrochronological analysis of the ship’s timber suggests a construction date of 1740–1750, although dates on either side of this timeframe are also considered possible. Additionally, pottery recovered from the site places the shipwreck early in the third quarter of the eighteenth century.

Successfully identifying BZN4 as*’t Hart* would provide the opportunity to explore trade and shipping between the Dutch Republic and its South American colonies in the eighteenth century from both an archaeological and a historical perspective.

**Fig. 2:**
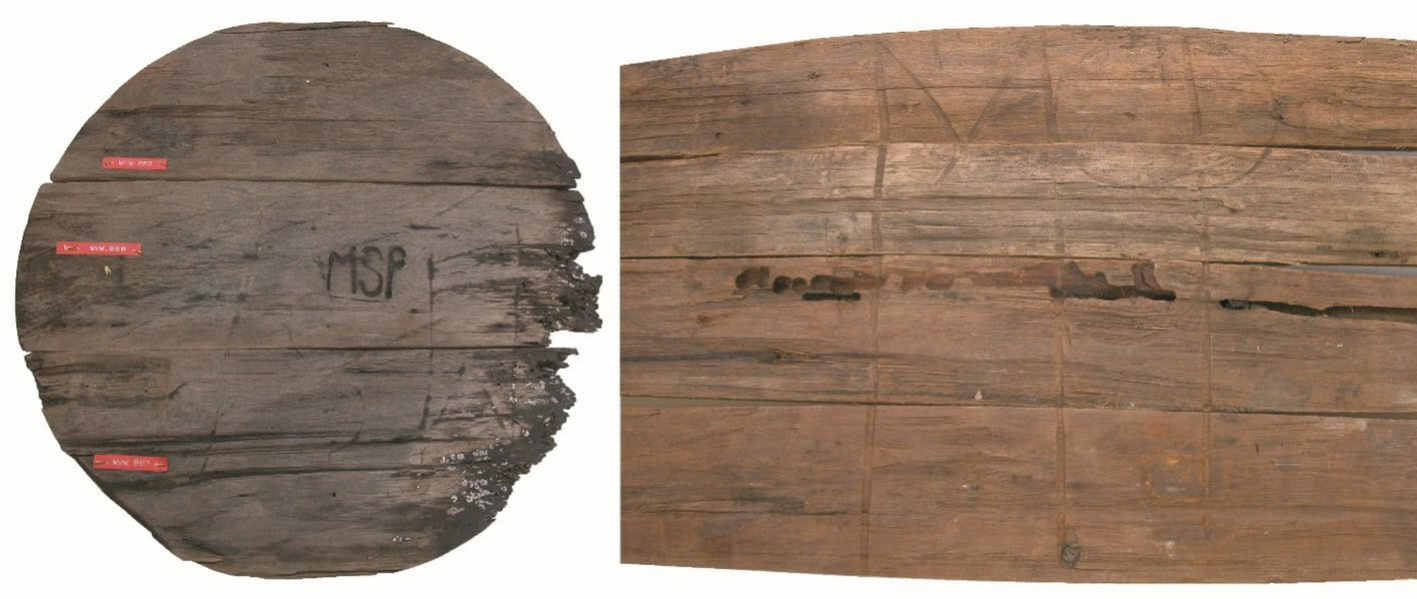
Two examples of the mark MSP

**Hypothesis** Identifying BZN4 as *’t Hart* was initially considered, because of the identification of several marks on the excavated casks, one of which consisted of the letters MSP; it was found four times on BZN4 (Fig. 2). This mark is that of Matheus Sigismundus Pallak (1709–1767), who owned several plantations in Suriname and was a member of the Hof van Politieke en Criminele Justitie (court). He was closely connected to Amsterdam merchant Arent Hartjes/Hartjens. Pallak represented Hartjens in Suriname, and Hartjens returned the favour, conducting business for Pallak in Amsterdam, including coffee sales.^2^

Arent Hartjens was the *boekhouder* (husband) representing the owners of the frigate*’t Hart*. The ship was built in 1739 and was sailing from Suriname, located on the northern coast of South America, when it sank just east of Texel on 26 November 1751. These details match the features of BZN4. Therefore, we propose that BZN4 is*’t Hart*.^3^

## Research Question

The main objective of this study is to assess whether it is possible to identify BZN4 as*’t Hart*. To address this, we ask two sub-questions:

- What details of the shipwreck BZN4 can be used to identify the ship?
- To what extent can the identification of BZN4 as*’t Hart* be justified?

## Research Methodology and Sources

### Methodology

The primary aim of this study is to determine whether BZN4 is*’t Hart*. The initial step involves identifying the characteristics of BZN4 that can be used to make this determination. The data from BZN4 serves as the starting point. However, since this shipwreck has only been partially excavated, not all of the ship’s features are known (see Sect. 3).

From the assessment of the archaeological data, the following details were selected:

- construction date
- ship’s dimensions
- ship’s use as a merchantman
- equipment and cargo on the final journey
- geographical location of the shipwreck
- date of wreck

We discuss the finding for each of these details in Sect. 4, first as they pertain to BZN4 and then to*’t Hart.* Our historical research focused solely on*’t Hart*, and we used various textual sources to garner information on the ship’s history. We then provide a comparative analysis of the overlap of the details.

### Sources

Archaeological data were gathered in several diving expeditions (Spoor-Hanraets and Jansma 1999; Spoor-Hanraets et al. 2001, 2002; Kleij 2002; Kuijper 2004; Den Braven 2006; Laarman and Lauwerier 2006; Kuijper and Manders 2009; Vos 2012). The data comprise various distinct features of the wreck, including its estimated construction date, ship dimensions, equipment, and cargo on her final journey, geographical location and estimated sinking date.

The textual sources pertaining *’t Hart* are found primarily in four collections:

1. Amsterdam notarial deeds

The Amsterdam Stadsarchief (SAA) holds the *aktes* (deeds) of the Oud Notarieel Archief (ONA).

There are three important deeds for researching *’t Hart*^4^:

*Declaratoir* = a declaration by a shipwright on behalf of the ship’s husband, stating that the ship was built in his shipyard, the date of its construction, and its measurements. During the uncertain period of the War of Austrian Succession (1740-1748), *declaratoirs* were used to prove the neutral status of Dutch ships. During wartime, a ship’s *declaratoir* was generally issued well after its date of construction, as is the case with *’t Hart*, which was issued in 1745, while the ship itself was constructed in 1739. The Amsterdam and the Zaandam archives hold many hundreds of such examples from the 1740s.

*Inventaris* = an appraisal of a person’s estate following death, listing assets and sometimes debts.

*Verklaring* = a statement, in this context of one or more crewmembers, commonly used to settle insurance claims. In the case of *’t Hart*, several crew members gave an account of the sinking of the ship on behalf of husband Arent Hartjens.

2. Archief van de Sociëteit van Suriname (SvS), held by the Nationaal Archief (NA), The Hague^5^

The plantation colony Suriname was governed by the Sociëteit van Suriname (Fatah-Black 2019), which levied an export duty of 2.5% on sugar, coffee, cocoa, cotton, lemon, gold, etc. Other products, notably dyewood (often used as stowage material) were exempt. The sources used in connection with the cargo of *’t Hart* relate only to the products that were taxed, not the entire cargo. For the most part, the different sources contain more or less the same information.

*Journaal Gouverneur* (governor’s daybook) = All incoming and departing ships in Suriname were listed. For the ships arriving, only the goods for the Sociëteit were noted. For the departing ships, the volume or weight of the taxed products was noted.

*Carga & Calculatie* = a list of products levied the 2.5% tax, probably prepared by a clerk. The 2.5% tax had to be paid in sugar, and the unit of account was one hogshead sugar (800 pounds [lbs]). The weight of the products being taxed had to be converted into a value of sugar: 1 lb coffee was valued at 6 lbs of sugar (later 10 lbs), and 1 lb cocoa was valued at 8 lbs of sugar. The list, as part of the *overgekomen brieven en papieren* (OBP; letters and papers dispatched), was sent to the Sociëteit directors in Amsterdam. An example from 1740 is shown in Table 1.

**Table 1:** The *Carga and Calculatie* of *’t Hart*, 1740

Cargo	Value	2.5%
	In sugar (lbs)	Sugar (lbs)
Sugar: 668.25 hogshead	534,600	13,365
Coffee: 74,275.00 lbs	445,650	1,114
Cocoa: 288.00 lbs	2,304	57
Bolleterie^*^: 10.00 lbs	400	10
		24,573 = 30.7 hogshead

*Massaranduba wood, used in windmill construction

*Source*: NA, SvS, no. 267/779 (OBP), 28 May 1740

**Table 2:** *Rekening* between captain Cornelis Roos and the Sociëteit, 1740 (guilders (ƒ) and sugar (lbs))

Taxed commodities		Debit			Credit		
		Value	2.5%				
		Sugar	ƒ	Sugar		ƒ	Sugar
Sugar	668.25 hogshead	534,6	668.0	13,365	Shipped for the SvS	57.0	1,14
Coffee	74,275.00 lbs	445,65	557.0	1,114	Idem	3.0	60
Cocoa	288.00 lbs	2,304	3.0	57	In cash (Holland)	1,163.5	13,273
Bolleterie^*^	10.00 lbs	400	0.5	10			
			1,228.5	24,573		1,228.5	24,573

*massaranduba wood, used in windmills

*Source:* NA, SvS no. 267/779 (OBP), 28 May 1740

*Rekening* = ship’s inventory of levied 2.5%: agreement between the ship’s captain and the Sociëteit, listing the cargo, the amount paid in sugar (debit) and the costs incurred by the Sociëteit (credit). The ship’s captain was responsible for paying the 2.5% tax to the Sociëteit. As a consequence, every ship leaving Suriname had a consignment of sugar for the Sociëteit on board. An example from 1740 is shown in Table 2.

*Factuur* = invoice for the hogsheads of sugar shipped for the Sociëteit. In 1741, for instance, 59 hogsheads (44,831 lbs) of sugar from eight plantations were shipped in *’t Hart*.^6^

- 3. Suriname Doop-, Trouw- en Begraafboeken (DTB, church records of christenings, marriages and burials). The NA in The Hague has digital copies, available on its webpage.
- 4. Suriname notary deeds (ONAS). The NA in The Hague has digital copies, available on its webpage.

## Research History of BZN4

BZN4 was first discovered in the Waddenzee, seven kilometers east of the Island of Texel in 1984 (Vos 2012, 169). From roughly the 16th to the 18th century, the deep waters to the east of the island were frequently used for anchorage by large seagoing vessels. Because these ships often had too deep a draft to sail fully loaded across the shallow Zuiderzee to ports like Amsterdam, Hoorn, and Enkhuizen, they anchored in the deep waters near Texel. Small boats and barges were then used to load and unload them as they waited for favourable winds to set sail.

The ships were usually safely sheltered behind the island, but occasionally disaster struck. During severe storms, multiple ships could be lost at once, sometimes even dozens in a single storm. Not all of these ships’ remains have been preserved on the seabed. However, since the 1960s and 1970s, remains of 75 to 100 ships have been found in the expansive area of the Texel roadstead.

### Burgzand Project

Burgzand is a relatively small, shallow area in the Texel roadstead (Vos 2012) where shipwrecks were first discovered in the 1970s and 1980s when fishermen’s trawl nets were being caught in places where they had never encountered obstructions before, due to shipwrecks emerging from the seabed. Recreational divers learned of these locations and discovered shipwrecks.

**Fig. 3:**
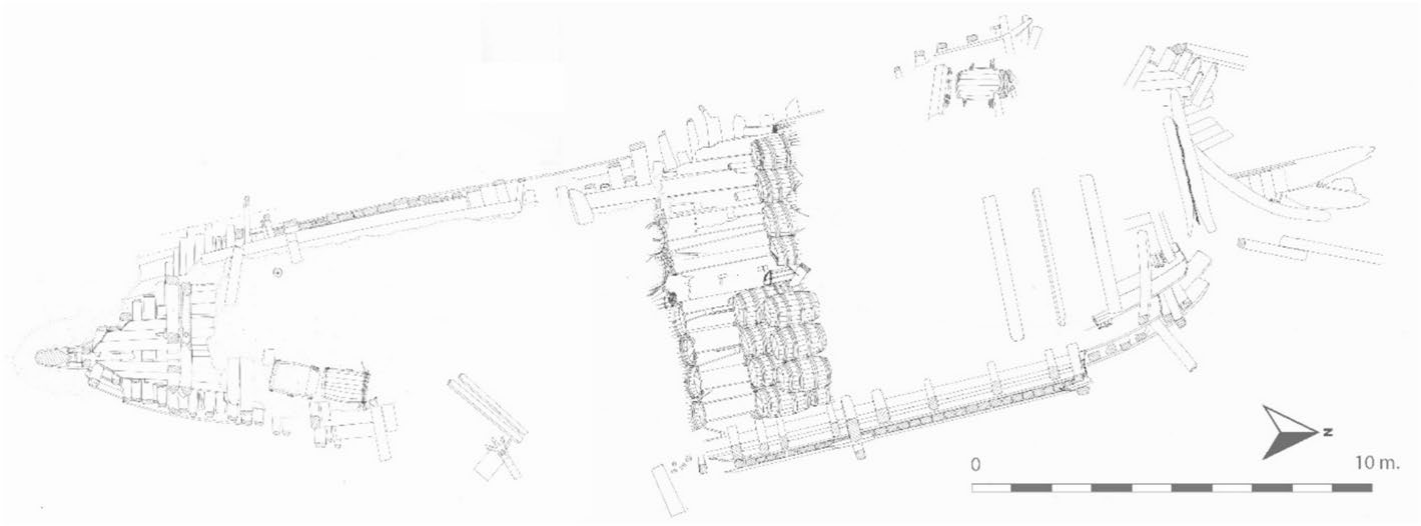
Aerial view of BZN4, showing the ship’s structure and cargo. *Source*: Vos 2012, 169, 373–374

In 1998, the professional archaeological dive team of the (former) Netherlandish Institute for Ship Archaeology (NISA), which was part of the Cultural Heritage Agency of the Netherlands, carried out archaeological research on Burgzand, focusing on an area approximately 1.5 km^2^ (Vos 2009, 2012). The goal was to document, date, and interpret wreck remains emerging from the seabed, in order to assess the research potential and identify the degradation processes affecting these remains (Vos 2005, 2012, 61–70) (Fig. 3).

**Fig. 4:**
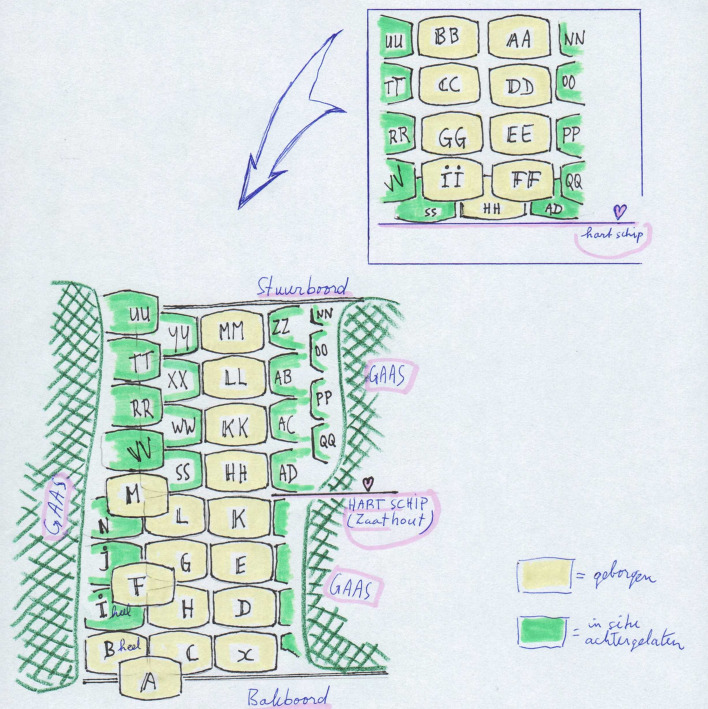
Overview of the midship trial trench. *Green*: casks left in the wreck. *Yellow*: casks recovered. *Source*: Vos 2012, 174

Between 1998 and 2005, the remains of 12 wrecks were documented. Additional research was conducted on some of the wrecks using exploratory trenches, and five shipwrecks were covered for protection using a new method involving scaffolding mesh (Vos 2012, 89–100) (Fig. 4).

### Burgzand Noord 4

One of these 12 wrecks in the Burgzand project was BZN4 (Vos 2012, 166–187). Local divers, who first explored the wreck around 1984 or 1985, described it as a small, not very seaworthy, open ship. Due to the presence of casks seemingly filled only with sand, which were therefore assumed to be water casks, the ship was believed to be a small lighter transporting fresh water from Texel to the ships moored in the roadstead, hence the alias *watervatenwrak* (water cask wreck; Vos 2012).

After the survey in 1999, additional archaeological research was carried out in 2001, involving three trial trenches at the rear, middle, and front of the ship (Fig. 4). This research provided a wealth of new information. The wreck site turned out to be much larger than initially anticipated. BZN4 stretched over an area of approximately 37 × 12 m and consisted of an almost complete bottom section from stem to stern and from port to starboard bilge (Fig. 3). About four meters of the sternpost was preserved and still stood upright. The stem had become dislodged and lay flat on the sea floor. No parts of the ship’s sides were found, apart from a small fragment on starboard.

The dimensions of the ship were estimated based on the remains, taking into account the different possible degrees for the stem’s rake and common length-to-beam ratios, which ranged from 4:1 to 4.5:1. With a beam of approximately 9 m, the length from stem to stern was estimated to be 35–37 m, although the keel was never fully exposed or measured during the archaeological investigation.

Dendrochronological samples from the ship’s construction were analyzed (Spoor-Hanraets and Jansma 1999; Spoor-Hanraets et al. 2001, 2002; Lückers 2002), resulting in estimated felling dates of 1742 ± 6–1744 ± 6. Taking into account the period between the felling of the trees and their use in the ship’s construction, the construction date was placed around 1740–1750, with a preference for the second half of the decade.

Based on the archaeological data, the sinking of BZN4 was dated early in the third quarter of the eighteenth century. This was primarily based on the ceramic assemblage (Kleij 2002) and the clay pipes in the wreck, which also indicated the ship had departed from a Dutch port (Den Braven 2006). Since the lifespan of early modern seagoing merchant ships was expected to be 15–25 years at most (Vos et al. 2019, 96–97, 112–119, 120–121), the sinking date aligns with the construction date (1740–1750).

Research was also conducted on the ship’s cargo, which primarily consisted of casks, 47 of which were documented underwater and 24 were recovered. The botanical samples from the casks consisted mainly of unroasted coffee beans, along with a few cocoa beans (Kuijper 2004; Kuijper and Manders 2011). In the heads of several casks, sugarcane stalks had been placed as a pressure valve in case the coffee beans fermented. The casks and their contents, along with the tropical timber used as stowage material, were the primary indicators that led to the identification of BZN4 as a West Indiaman, transporting goods from one of the Dutch West Indian colonies to the Netherlands (Vos 2012).

## Identification of BZN4 as*’t Hart*

The identification of BZN4 as*’t Hart* is based on several distinct details of the ship, which we present in chronological order. First, the construction and the dimensions of the ship will be examined. Subsequently, its use as a merchantman will be discussed. And finally, its final journey, detailing the equipment and cargo on board. Each section addresses these details as evident in BZN4, followed by the parallel specifics of*’t Hart*, and concluding with a comparison of the two.

## Construction Date

### BZN4

Based on the results of the archaeological investigation, the construction date of BZN4 was estimated to be between 1740 and 1750 (Vos 2012, 187). This timeframe was established through a dendrochronological analysis of eight samples extracted from the ship’s hull, which were considered parts of the original ship and not of subsequent repair work.

Three of the samples, which had sapwood on them, provided felling dates of 1742 AD ± 6, 1743 AD ± 6, and 1744 AD ± 6 (see Table 3). However, it should be noted that the margin of ± 6 years allows for some flexibility in this dating. Besides the dendrochronological datings, the estimation of the construction date is also based on the time required for the timber to be transported from the felling site to the shipyard. The comparative analysis with*’t Hart* calls for a critical review of the dendrochronological dating and the uncertainties associated with it.

**Table 3:** Summary of the dendrochronological samples from BZN4

Find nr	Estimated Felling Date	Date of Tree Rings	# of Tree Rings	# of Sapwood Rings	Provenance	Description
WVW-518	post-1734 ± 6	1599–1704	106	–	Central Germany	Unidentified beam from original ship
WVW-519	1743 ± 6	1638–1728	91	5	Germany, Schleswig-Holstein	Unidentified beam from original ship
WVW-1480	1742 ± 6	1610–1730	121	8	Germany, East Frisia	Rider
WVW-1830	post-1700 ± 8	1510–1672	163	–	Germany, East Frisia	Frame timber
WVW-1833	1744 ± 6	1627–1736	111	12	Germany, East Frisia	Frame timber
WVW-1762	post-1692	1566–1688	123	–	Germany, Hannover	Frame timber
WVW-1831	post-1732	1628–1718	91	–	Central Germany	Rider
WVW-1832	post-1735	1611–1721	111	–	Central Germany	Frame timber

*Source:* Spoor-Hanraets and Jansma 1999; Spoor-Hanraets et al. 2001, 2002

### *’t Hart*

In 1739, Arent Hartjens purchased the bespoke two-deck frigate from Zaan shipwright Adriaan Jacobsz Ouwejan (1685–1749). In November that year, the ship set sail for Suriname under captain Cornelis Roos.^7^

Ouwejan belonged to a well-known shipbuilding family. In his day, he was one of the major Zaan maritime entrepreneurs. Commencing as an apprentice at age 15, he was a skilled shipwright within a decade. His first recorded ship sale dates back to 1708, marking the beginning of a prosperous enterprise. As years elapsed, he expanded his domain, amassing three shipyards, a timberyard, and a house. By 1749, his estate exceeded ƒ40,000, including shares in 17 ships and *vleten* (whaling gear in the Zaan region).^8^

Ouwejan is thought to have constructed more than 100 ships, of which 88 have been identified. Among the ships identified were 55 *fluitschepen* (fluyts) (62.5%) and 16 frigates (18.2%) including*’t Hart*. The average dimensions of the frigates built by Ouwejan were 105.8 × 29.0 × 12.5 × 5.6 ft (N = 12). Two stand out: a 124-foot ship and one with a *gewrongen spiegel* (sloping transom), a British design.^9^

Since the latter half of the seventeenth century, Zaan shipwrights used predominantly oak from the Rhineland. The logs were floated down the Rhine and sawn at Amsterdam and Zaan windmills. But following the Great Northern War (1700–1721), the trade of Baltic timber resumed. In addition, the timber imports from North Germany increased over the course of the eighteenth century. This pre-sawn Hamburger timber came from the Weser and Elbe River basins. This oak was heavier than the Rhinish, but of inferior quality (Schillemans 1947; Adam 2015; Enthoven 2023). At the local timber auctions, Ouwejan purchased oak beams and arched timbers in 1738/39, both originating from the Rhineland or Hamburg (North Germany). This timber was likely used in the construction of*’t Hart*.^10^

Arent Hartjens was the son of lawyer Jacob Hartjens. Arent’s estimated inheritance was around ƒ300,000. It is apparent that he was active in the Suriname trade as early as 1731. As the ship’s husband, he ordered from Adriaan Ouwejan the 91-foot frigate *Westerveen*, named after his wife Margaretha Westerveen, in 1736, to be followed by*’t Hart* three years later. The *Westerveen*, as*’t Hart*, made several voyages to and from Suriname. Hartjens did not pursue a political career, but became a colonel in the civic militia of Amsterdam. Until his death on 26 July 1779, he was involved in the Suriname trade. He owned a spacious townhouse on the Singel between the Brouwersgracht and the Blauwburgwal where, in the *pakzolder* (attic), coffee was sorted, weighed and repacked. He owned large shares in the plantations Laarwijk and Belgard (both on the Commewijne River), a house in Paramaribo and 20 enslaved Africans. At the time of his death, his assets were ƒ194,691 and debts ƒ131,101. In total, his estate was worth only ƒ63,590. He must have been a particularly unsuccessful entrepreneur, or possibly he squandered his money on gambling, booze and women. He never remarried after his wife’s death in 1760 (Zandvliet 2020, no. 55).^11^

### Comparative Analysis

*’t Hart* was constructed in 1739 and sailed to Suriname in November of that year.^12^ A total of eight BZN4 samples have been successfully dated dendrochronologically. Fragments of sapwood were detected on three samples, facilitating the estimation of felling dates based on the missing sapwood rings. This analysis yielded estimated felling dates of 1742, 1743, and 1744, with a 1ẟ margin of six years. These dates create a tight timeframe relative to the start of the construction of*’t Hart* in 1739. Therefore, these dendrochronological estimates have been reassessed.

The dendrochronological analyses were conducted by RING between 1999 and 2002, making them at least 22 years old (Spoor-Hanraets and Jansma 1999; Spoor-Hanraets et al. 2001, 2002). The 1ẟ margin for the original estimated felling dates only covers 68.3% of cases, meaning there is a 31.7% chance the actual felling date falls outside the interval. Additionally, this method only considers single samples and does not use the additional information that multiple overlapping estimated felling intervals provide.

**Fig. 5:**
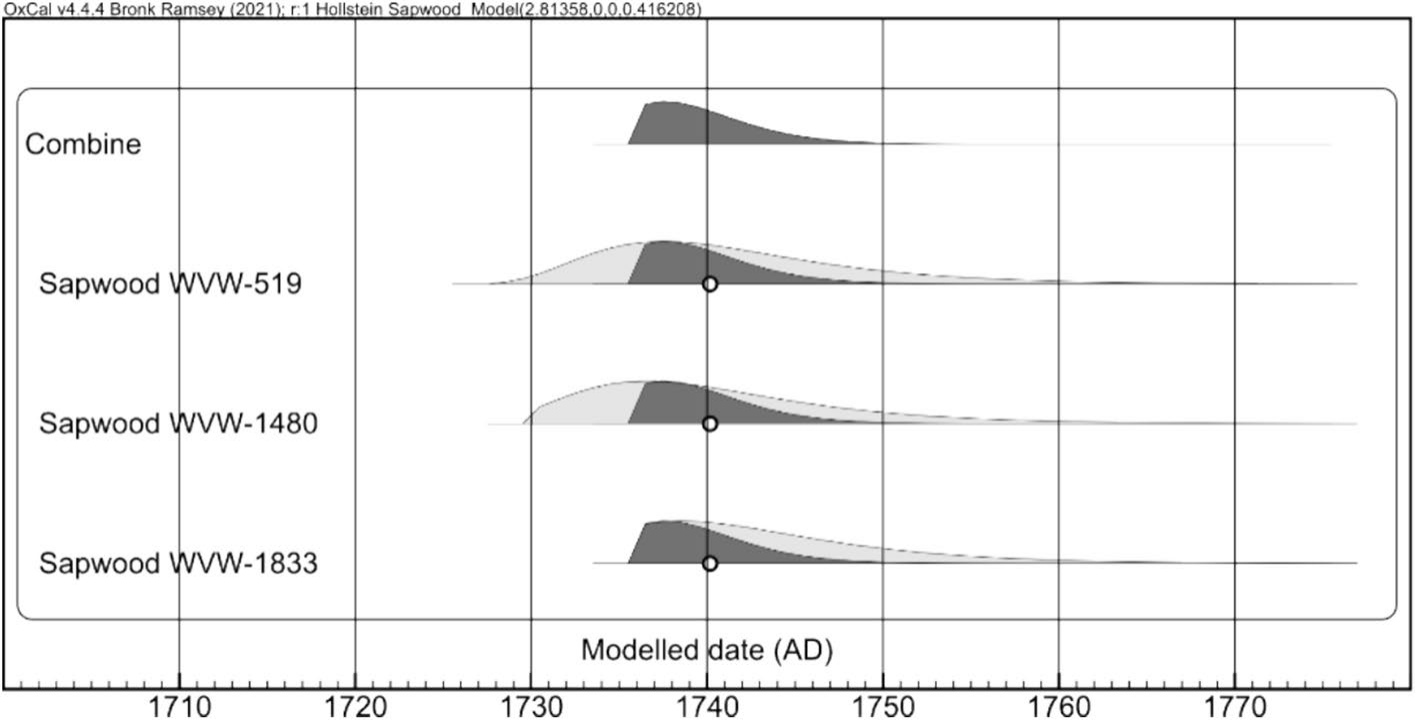
Probability density distribution for the three samples of sapwood fragments (*light grey*) and the combined felling interval (*dark grey*)

The estimated felling dates were recalculated using Bayesian statistics (Table 4; Fig. 5; Bronk Ramsey 2009). A new, combined probability distribution was derived from an individual probability distribution with an estimated felling date and a 95.4% confidence interval (Fig. 5). The difference in the estimated felling interval obtained in 1999–2002 (1738–1748) and those obtained using our method (1736–1746) is minimal. However, it is not clear from the initial reports what the accuracy of this estimate is.

**Table 4:** Reassessment of dendrochronological samples with sapwood from BZN4 Using the Hollstein Sapwood model

Find #	Estimated felling date (1999–2002 reports)	μ	Beginning date	End date	Estimated felling date (2025 evaluation)
WVW-519	1743 AD ± 6	1741	1729	1757	1741 (1729–1757)
WVW-1480	1742 AD ± 6	1741	1730	1756	1741 (1730–1756)
WVW-1833	1744 AD ± 6	1744	1736	1759	1744 (1736–1759)
Combined		1740	1736	1746	1736–1746

The combined felling interval of the dendrochronological samples of BZN4 was placed around 1740, with a 95.4% confidence interval from 1736 to 1746. As with the individual estimates, this is nearly identical to the earlier intervals and fits the known construction year of 1739. For samples without sapwood, only the earliest possible felling year can be estimated. As expected, these fall some years before 1739. However, the notation used for these dates in the initial reports stated both a lower boundary (i.e., *after* year x) as well as an interval (i.e., x ± 6), leaving room for multiple interpretations of the felling interval.

Initially, three dendrochronological samples containing sapwood posed a challenge in identifying BZN4 as*’t Hart*. These samples provide an estimated felling year that postdated the construction year of 1739, if the margin is not taken into account (Hollstein 1980). However, it should be noted that sapwood estimates are derived from large populations that are then applied to individual trees. The number of sapwood rings (and therefore any estimates based on them) vary based on age (Hollstein 1980) and provenance (Haneca 2005), but also between trees (and even in a single tree) as part of natural variation. Furthermore, adding or removing samples can shift the resulting estimated intervals (Bridge 2013). Variations of a small number of years between estimated felling years and the construction year such as this case should not be taken as an argument to reject the identification of BZN4 as*’t Hart*.

## Ship Dimensions

### BZN4

The estimated length of the wreck from stem to stern was approximately 35–37 m. This measurement was determined without the keel being measured, as it is inaccessible lying deep beneath the shipwreck’s structure, but is based on the ship’s beam of approximately 9 m and an average length-to-beam ratio of about 4:1 for Dutch ships in the eighteenth century. As mentioned above, the sternpost was found in position, but the stempost was lying flat on the seabed. The width of 8 m high up on the bilge, suggested a beam of about 9 m. The remaining part of the stern was approximately 4 m high. The skin planks and ceiling planks were made of oak, approximately 7 and 5 cm thick. The tops of the frame timbers were too eroded to measure reliably, but they were at least 13 cm thick. Deeper in the ship’s structure, where less erosion had occurred, the cross-section of the frame timbers measured between 30 and 35 cm. Outside the ship, pine doubling planks with a layer of animal hair and tar, intended as protection against naval shipworms (*Teredo navalis*), were found.

All these details indicate a medium-to-large seagoing ship intended for tropical destinations where naval shipworms were present (Vos 2012, 166–187).

### *’t Hart*

In the ship’s *declaratoir*, the following dimensions for the two-deck frigate*’t Hart* are given:

- length from stem to stern: 106 *voet* and 3 *duym* (30.8 m),^13^
- beam: 29 *voet* and 7 *duym* (8.4 m),
- hold: 12 *voet* and 9 *duym* (3.6 m),
- deck: 5 *voet* and 2 *duym* (1.5 m).

Additionally, the ship had a half deck and a forecastle.^14^

The Zaan region just north of Amsterdam was the largest maritime pre-industrial area of Europe. Around 1730, hulls were being built on docks in 38 shipyards, supported by dozens of timberyards, sawmills, ropewalks, mast and tackle makers, and anchor forgers (Enthoven 2018a).

Of the ships built in the Zaan region, almost three quarter were *fluitschepen* (fluyts) (Table 5). A Zaan specialty was medium-sized fluyts of 110–115 ft, built on venture without a commission. They began as whalers, to be sold later. Frigates were in general ordered and purposely built, like the*’t Hart*. In the 1740s, the dimensions of an average Zaan frigate were 105 × 29 × 12.5 × 5.2 ft (N = 36), with a ratio of 3.6:1 (Enthoven 2018b, 2020).

**Table 5:** Ships Sold in Zaandam, 1701–1750, and Dimensions of Ships Around 1700 (in ft, N = 815)

Ships Sold in Zaandam, 1701–1750			Average Specifications Dutch ships					
Type	No	%	*Last*^†^	Length	Beam	Hold	Deck	Ratio
Fluyt	579	71.0	213	129	29.0	13.0	5.25	4.4 : 1
*Katschip*	14	1.7	150	123	23.5	13.0	-	5.2 : 1
*Hekboot*	27	3.3	248	117	33.0	14.5	6.80	3.5 : 1
Frigate	59	7.2	196	109	32.5	12.5	5.50	3.4 : 1
*Hoeker*	18	2.2	116	90	27.0	12.0	–	3.3 : 1
*Galjoot*	68	8.3	85	84	25.0	11.0	–	3.4 : 1
*Boeier*	–	–	73	83	21.0	10.5	–	4.0 : 1
*Kofschip*	5	0.6	69	81	22.0	9.7	–	3.7 : 1
*Smakschip*	3	0.4	36	68	19.0	7.0	–	3.6 : 1
Not specified^*^	42	5.5						
Total (N)	815	100.0						

*Predominantly inland ships

†1 *last* = 2,000 kg or 2 metric ton

*Source*: Enthoven dataset (Zaan ships sold); SAA, Archief van de Directie van de Oostersche Handel en Reederijen no. 244, 1688 (average specifications Dutch ships)

**Table 6:** Voyages of*’t Hart* Between Suriname and Amsterdam, 1740–1751

ID	Captain	Suriname		Sugar		Coffee	Cocoa
		Arriving	Departing	Hogshead	lbs	lbs	lbs
1267.0	Roos, Cornelis	27-Feb-1740	1-Jun-1740	668	534,400	74,275	288
1306.0	Roos, Cornelis	14-Feb-1741	20-May-1741	657	525,600	77,235	318
1363.0	Roos, Cornelis	9-Jan-1742	29-Apr-1742	737	589,600	23,429	0
1411.0	Roos, Cornelis	8-Dec-1742	19-May-1743	742	593,600	26,388	261
1471.0	Roos, Cornelis	Unknown	14-Nov-1744	741	592,800	20,895	2,667
1546.0	Verbrugge, Dirck	13-Nov-1745	26-Jul-1746	730	584,000	36,997	7,732
1601.0	Verbrugge, Dirck †	13-Mar-1747	21-Oct-1747	607	485,600	89,334	6,248
1709.0	Orelius, Pieter	26-Oct-1749	21-Feb-1750	654	523,200	45,149	2,846
1793.0	Orelius, Pieter (disqual)	29-Mar-1751	30-Aug-1751	619	495,200	74,204	6,586
Average*’t Hart*				684	547,111	51,990	2,994
Total Suriname export, 1740–1749					177,053,800	32,668,847	3,280,600
Average per ship (N = 444)					400,000	73,578	7,389

*Source*: Postma Suriname Dataset, available from DANS, https://dans.knaw.nl; Postma 2003; NA, SvS no. 267/779 (OBP), 28 May 1740; SvS no. 268/716 (OBP), 18 May 1741; SvS no. 268/717 (OBP), 18 May 1741; SvS no. 268/728 (OBP), 20 May 1741; SvS no. 269/825 (OBP), 27 April 1742; SvS no. 271/846 (OBP), 17 May 1743; SvS no. 271/857 (OBP), 18 May 1743; SvS no. 199/176, 17 May 1743; SvS no. 274/583 (OBP), 15 November 1744; SvS no. 274/625 (OBP), 16 November 1744; SvS no. 200/310, 26 July 1746; SvS no. 200/597, 20 October 1747; SvS no. 201/396, 21 February 1750; SvS no. 202/73, 30 August 1751

**Fig. 6:**
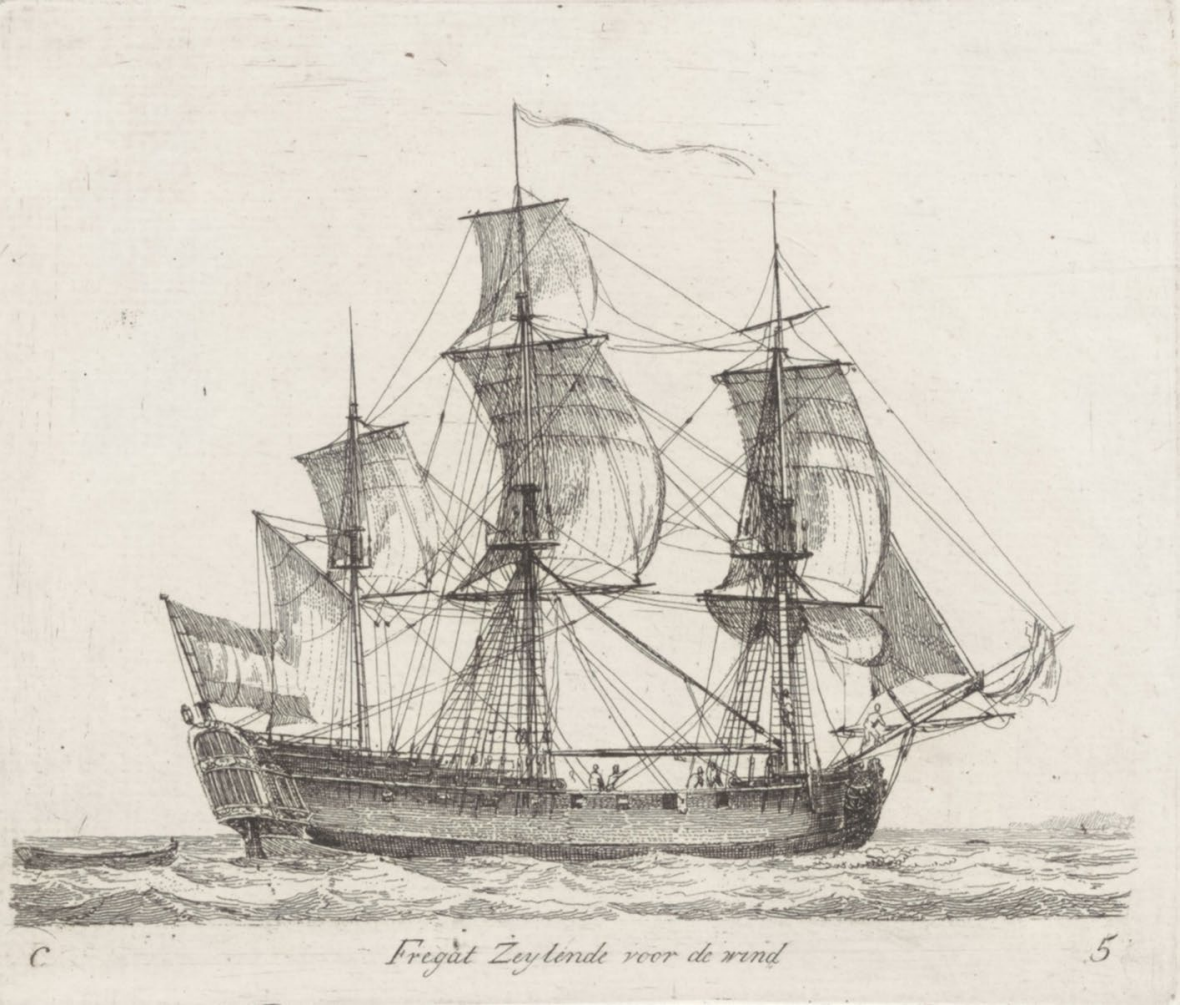
A frigate sailing before the wind. Engraving by Gerrit Groenewegen, 1789. *Source*: Zuiderzeemuseum no. 003965

A frigate was a small to large merchantman with a square stern and a V-shaped bottom that rose sharply in the stem and stern (Fig. 6). The rake or overhang of the sternpost extended rather backward. They were nicknamed *hanggat* (lazy ass) and built for speed rather than cargo capacity. They were usually armed, hence the large ratio of 3.4:1 (Enthoven 1991, 2013). With a length of 106 ft (30.8 m), the*’t Hart* was a small merchantman, but an average frigate.

### Comparative Analysis

Based on the archaeological data, the initial estimates of the dimensions of BZN4 were different than those of the*’t Hart*. The original estimated length of BZN4 was 35–37 m, significantly longer than the documented length of*’t Hart*, which was 30.8 m, but BZN4’s estimated maximum beam of approximately 9 m closely matches*’t Hart*’s beam of 8.4 m. This discrepancy in length provided a reason to reassess the estimated dimensions of BZN4.

The estimated beam of 9 m for the BZN4 beam was reliable since both sides of the ship’s bottom have remained relatively intact. The length of BZN4 was harder to estimate accurately, because of the collapsed stem and the uncertainty of the steepness, or rake, with which it had been positioned in the ship’s structure. Therefore, the initial estimation of the length of the ship relied on an average length-to-beam ratio of approximately 4:1. Taking into account a margin of error, an estimated length of 35–37 m for BZN4 was established.

However, the average length-to-beam ratio of Dutch frigates during this period is 3.4:1, which sets them apart from other contemporary ships (Table 5). Using this ratio, the length of BZN4 can be determined to be approximately 30.6 m, which closely matches the documented length of*’t Hart* at 30.8 m.^15^ The reconstruction of the main dimensions of the ship’s remains, therefore no longer serve as a contra-indication for identifying BZN4 as*’t Hart*.

## Use as a Merchantman

### BZN4

Evidence of a wreck’s previous use is difficult to identify archaeologically. This is especially true for BZN4, because it was only partially excavated and because of the extensive deterioration of the site. This means that the archaeological research has not yielded additional information on its use. However, the archaeological data provide enough material to characterize the ship’s general use, namely, that it was in service as a merchantman and not as a (heavily built) warship or a fishing vessel.

### *’t Hart*

*’t Hart* made nine voyages to and from Suriname (Table 6). On the outgoing leg, the ship carried mainly provisions for the Sociëteit and the privately owned plantations. This included cereals, salted meat and building materials like chalk, cement and bricks for plantations. The ship also carried passengers, mostly Sociëteit soldiers. It was a typical *Surinamevaarder* (Suriname ship).^16^

Cornelis Roos captained the first five voyages. During his last trip,*’t Hart* briefly got stuck on a mudbank at the estuary of the Suriname River. Dirck Verbrugge captained the next two trips. He died in 1747. Claas Grell took Verbrugge’s place, running into trouble with a French privateer on the return trip. The French confiscated part of the cargo. Captain Pieter Orelius sailed the ship’s second-to-last voyage and was also the captain as the ship began its last journey. But after departing from Texel in 1751, the foremast broke, requiring the crew to stop at Plymouth for repairs. Orelius succumbed to mental illness while there and was succeeded as captain by first mate Pieter Groenenberg. Groenenberg captained the ship on its last voyage when it sank east of Texel on 26 November 1751. Groenenberg survived but never sailed to Suriname again.^17^

### Comparative Analysis

Due to the limited archaeological data on the use of BZN4 as a merchantman, a comparison with*’t Hart* provides little additional information. However, based on descriptions of*’t Hart*’s previous voyages as a merchantman, a comparison can be made between the registered cargo weight of its final voyage and that of earlier voyages. This comparison reveals that the total cargo weight of the last voyage, 575,990 lbs, was below the average of 602,095 lbs recorded for*’t Hart*. The total registered cargo weight on*’t Hart* was not excessive and is unlikely to have influenced the ship’s stability on its last voyage.

## Equipment and Cargo on the Final Journey

### BZN4

Materials recovered from BZN4 can be interpreted as the ship’s inventory, provisions and cargo. The inventory consists of a limited number of artifacts, with 37 finds allocated to pottery and five to artifacts of glass. Additionally, the following items were found on BZN4: 12 animal bones, 34 metal objects, nine fragments of clay pipes, and two flintstones (Vos 2012, 181–183).

The analysis of the pottery indicated a dating early in the third quarter of the eighteenth century and that it originated from the Netherlands and the German Rhineland. In terms of composition, this assemblage does not differ from other contemporary Dutch sites, leading the researchers to designate BZN4 as a Dutch ship.

Another category of finds on BZN4, clay pipes, can be accurately dated. Out of the nine fragments, only four were deemed to be part of the inventory of BZN4 (Den Braven 2006).^18^ The other five likely were embedded in the ship some time after the ship had foundered, as their dating differs.^19^ Among the four inventory fragments, only one bowl was found. Of the other three clay pipes, only the stems were preserved. The bowl was oval-shaped with a mark bearing the initials VD.^20^ This pipe was probably manufactured in Gouda by either Klaas Marté (1733–1782) or his widow, Trijntje Cornelis Pot (1782–1788).^21^

The ship’s inventory also included a fake wooden cannon barrel, which probably had been used previously for its deterrent value but served as stowage material on BZN4. Additionally, cooper’s tools such as a short-handled axe were found. Some of the staves recovered were interpreted as processing waste, making it possible that a cooper had been on board who made or repaired casks (Vos 2012, 172).

The vast majority of the artifacts found on BZN4 were located in the hold and designated as cargo (87%). Stowage material made from tropical hardwood had been placed under the casks to create a level surface. During the exploratory trench investigation in 2001, at least 47 casks were documented, of which 24 were recovered (Vos 2012). It is certain that many more casks were on board BZN4. The casks contained mainly remnants of unroasted coffee beans and several cocoa beans (Kuijper 2004; Kuijper and Manders 2011).

**Fig. 7:**
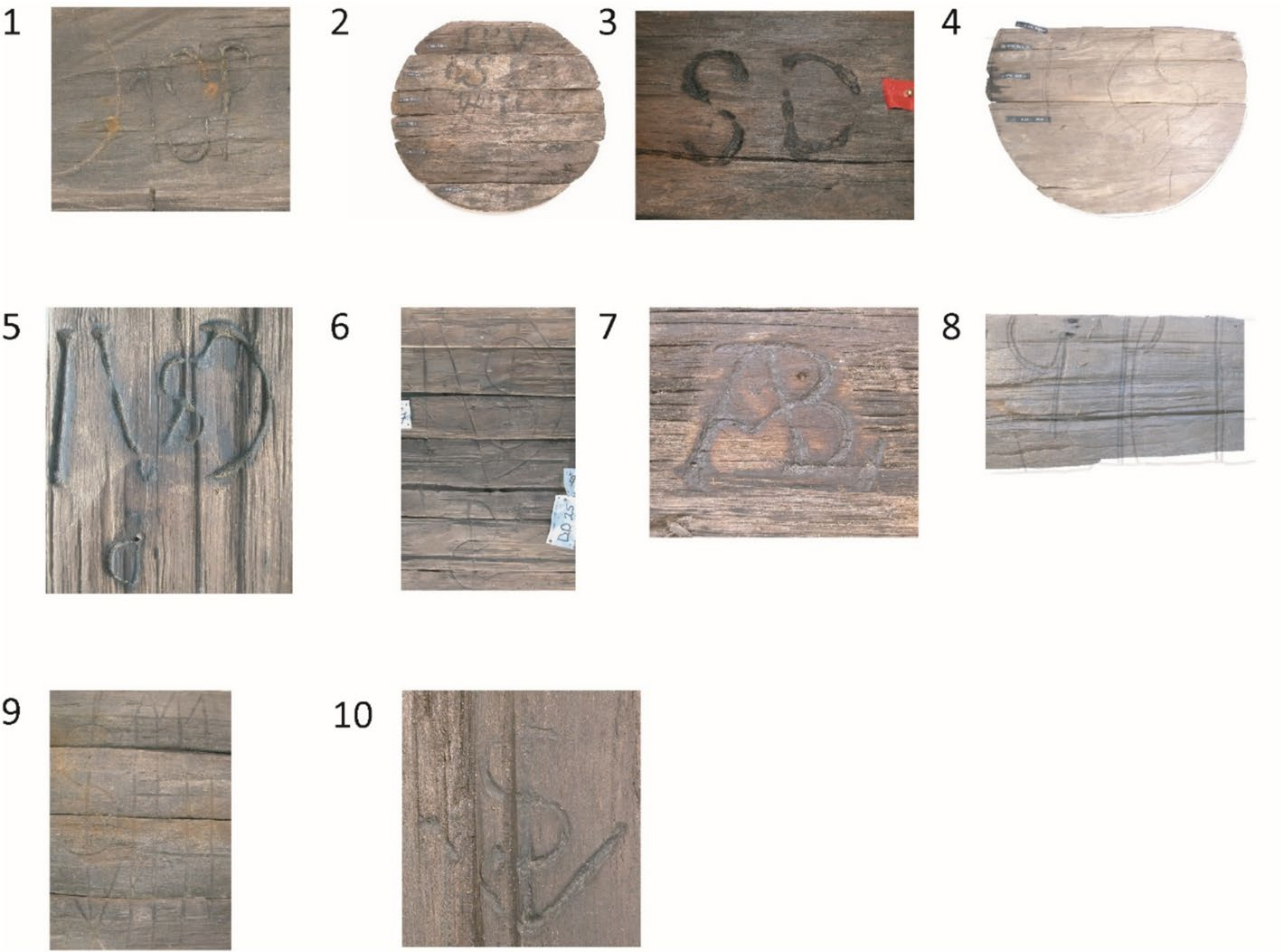
A selection of the marks found on casks on BZN4

Timber species and origin were determined for 79 wood samples, revealing 21 different timber species. For 15 of these, the genus or even the species could be identified. Of these, 14 are found in the tropical northeast of South America: Venezuela, Guyana, Suriname, French Guiana, and Brazil. For six species, neither genus nor species could be identified, but the wood anatomy in all cases indicated a tropical origin (Vos 2012, 184–185). Two hundred and twenty-two marks were found on the casks from BZN4, of which about 42 are unique. The precise number of unique marks is difficult to estimate because of their fragmentary preservation. Many consist of letter combinations, monograms, and illustrations (Fig. 7; Table 7). A significant portion of the marks could be identified and could be matched with merchants and plantations.

**Table 7:** Cask marks found on BZN4 that could be connected to a person

Number	Mark	Person identified	Product^*^
1	MSP	Matheus Sigismundus Pallak	Coffee
2	IPV SD	Jan Pieterz Visser and his wife Anna Dufour	Coffee and cocoa
3	SD	The plantation Sardam, planter Jan Pieterz Visser and wife	Coffee and cocoa
4	ECS	Ephraim Comans Scherpingh	Not sampled
5, 6	IVSD	Jan van Sandick, later Jean Couderc^†^	Coffee
7	ABL	Jan Labadie	Not sampled
8	GPI	Ephraim Comans Scherpingh	Unknown
9	Warehouse	Johan van Scharphuizen, later the heirs of Jonas Witsen	Coffee
10	Anchor S	Johan van Scharphuizen, later the heirs of Jonas Witsen	Coffee

*The product was determined through the samples that were taken during the archaeological research

†A record from 1743 mentions Jean du Peirou/Pérou with this mark transporting sugar to Isaac Couderc. Jean du Peirou was administrator of Van Sandick’s plantation Roosenburg in Suriname. Couderc was a merchant and executor of the Roosenburg estate after Van Sandick’s death. SAA, ONA no. 10036/232, 12 May 1744; ONA no. 10432/2017, 1 October 1750. For Roosenburg, see Oostindie 1989

*Source:* NA, ONAS no. 760/439, 15 August 1747; ONAS no. 760/721, 16 February 1752; ONAS no. 760/724, 27 August 1752; SAA, ONA no. 9766/35, 14 May 1749; no. ONA 10430/1601, 5 August 1750; NA, ONAS no. 760/758, 26 February 1752; ONAS no. 760/723, 17 February 1752; NA, SvS no. 213/194, 5 March 1685; NA, ONAS no. 759/72, 30 July 1740; ONAS no. 761/288, 13 June 1758; no. 761/126, 23 April 1756

**Table 8:** Overview of the 161 casks stored beneath the orlop deck

Number in Fig. 8	Mark	Person identified	Cargo		
			Casing	Number of casks	Product
1	SDP	Miss J. M. Duplessis	Cask	6	Cocoa
1	SDP	Miss J. M. Duplessis	Cask	10	Coffee
2	IPV SD	Anna Dufour, widow of J.P. Visser SD = plantation Sardam	Hogshead	40	Sugar
3	ECS	Ephraim Comans Scherpingh	Hogshead	10	Sugar
4	GPI	Ephraim Comans Scherpingh	Hogshead	20	Sugar
5	IH	Ephraim Comans Scherpingh	Hogshead	10	Sugar
6	CK	Elias van der Gaegh	Cask	5	Coffee
7	IB	Widow of A. Braet	Hogshead	10	Sugar
8	ABL	Jan Labadie	Hogshead	15	Sugar
	Unclear	Jan Hendrik Muller	Hogshead	18	Sugar
9	HTBS	Nicolaas Lemmers	Hogshead	17	Sugar
Total				161	

*Source*: NA, ONAS no. 760/720, 16 February 1752; ONAS no. 760/721, 16 February 1752; ONAS no. 760/723, 17 February 1752; ONAS no. 760/724, 17 February 1752; ONAS no. 760/725, 17 February 1752; ONS no. 760/740, 24 February 1752; ONAS no. 760/758, 26 February 1752; ONAS no. 760/765, 13 March 1752; ONAS no. 760/767, 10 April 1752

### *’t Hart*

**Fig. 8:**
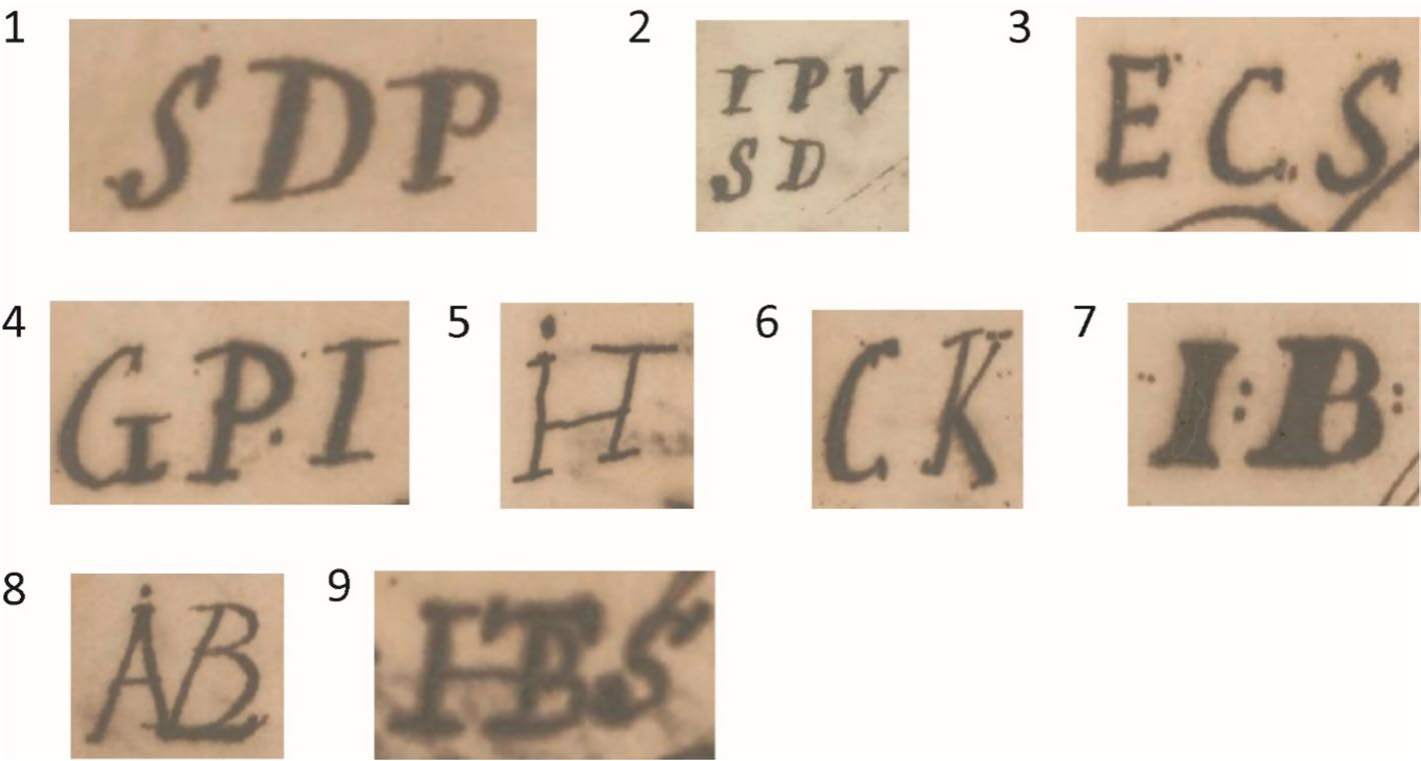
Marks on the 161 casks stored beneath the orlop deck of *’t Hart*

*’t Hart* left Suriname on what would be its last voyage with 619 hogsheads of sugar (15 for the Sociëteit), 74,204 lbs of coffee, and 6,585 lbs of cocoa.^22^ Receipts of the cargo also list 161 casks stored beneath the orlop deck (Table 8; Fig. 8).

### Comparative Analysis

The identification of BZN4 as*’t Hart* is primarily based on the matching cargo of the two.*’t Hart* transported 619 hogsheads of sugar, 74,204 lbs of coffee and 6,585 lbs of cocoa on its final journey. Unroasted coffee beans, along with a few cocoa beans, were found in the casks of BZN4. Additionally, casks were found on BZN4 that appeared empty and had been identified as water casks during the initial archaeological research. But the original contents of coffee or cocoa may have been washed out of the casks, or the sugar they contained may have dissolved over the centuries.

A match can also be made in terms of the quantity of cargo. To calculate the total number of casks on board, the weight of the coffee and cocoa must be converted into the equivalent number of casks. A crucial factor in this calculation is estimating the weight contained in an individual cask. Based on the dimensions of the recovered casks, we calculated an average capacity of 330 L.^23^ The specific weight of coffee and cocoa beans was 750 kg/m^3^, or 1,518 lbs/m^3^.^24^ The 6,585 lbs of cocoa and 74,204 lbs of coffee on board is approximately the equivalent of 4.34 m^3^ of cocoa and 48.88 m^3^ of coffee. Converted into casks with a capacity of 330 L, this amounts to 13 casks of cocoa and 148 casks of coffee. Combined with the registered number of hogsheads of sugar, this brings the total of estimated cargo casks on*’t Hart* to 780.

To estimate whether this is a realistic number of casks to be transported on*’t Hart*, the total tonnage of a mid-eighteenth century frigate was considered. The registered cargo on*’t Hart* weighed a total of 258 metric ton (780 casks of 330 L each). On average, a frigate measuring 109 ft had a capacity of 196 *last* (390 metric ton, Table 5). Given that*’t Hart*, at 106 ft, could accommodate approximately 190 *last* (380 metric ton), there would have been ample space for additional commodities.

The marks on*’t Hart*’s registered cargo and those on the cargo found on BZN4 were also compared. It should be noted that the data from both archaeological and textual sources are far from complete. Only 47 casks from BZN4 were documented, representing just 3% of the estimated original shipment of at least 780 casks. Similarly, the textual sources provide insight into only a small number of the marks. While the cargo manifest lists a total of 780 casks on board*’t Hart* (Table 1), only 161 casks were recorded in the cargo list (Table 8), meaning that markings were recorded for just 21%.

**Fig. 9:**
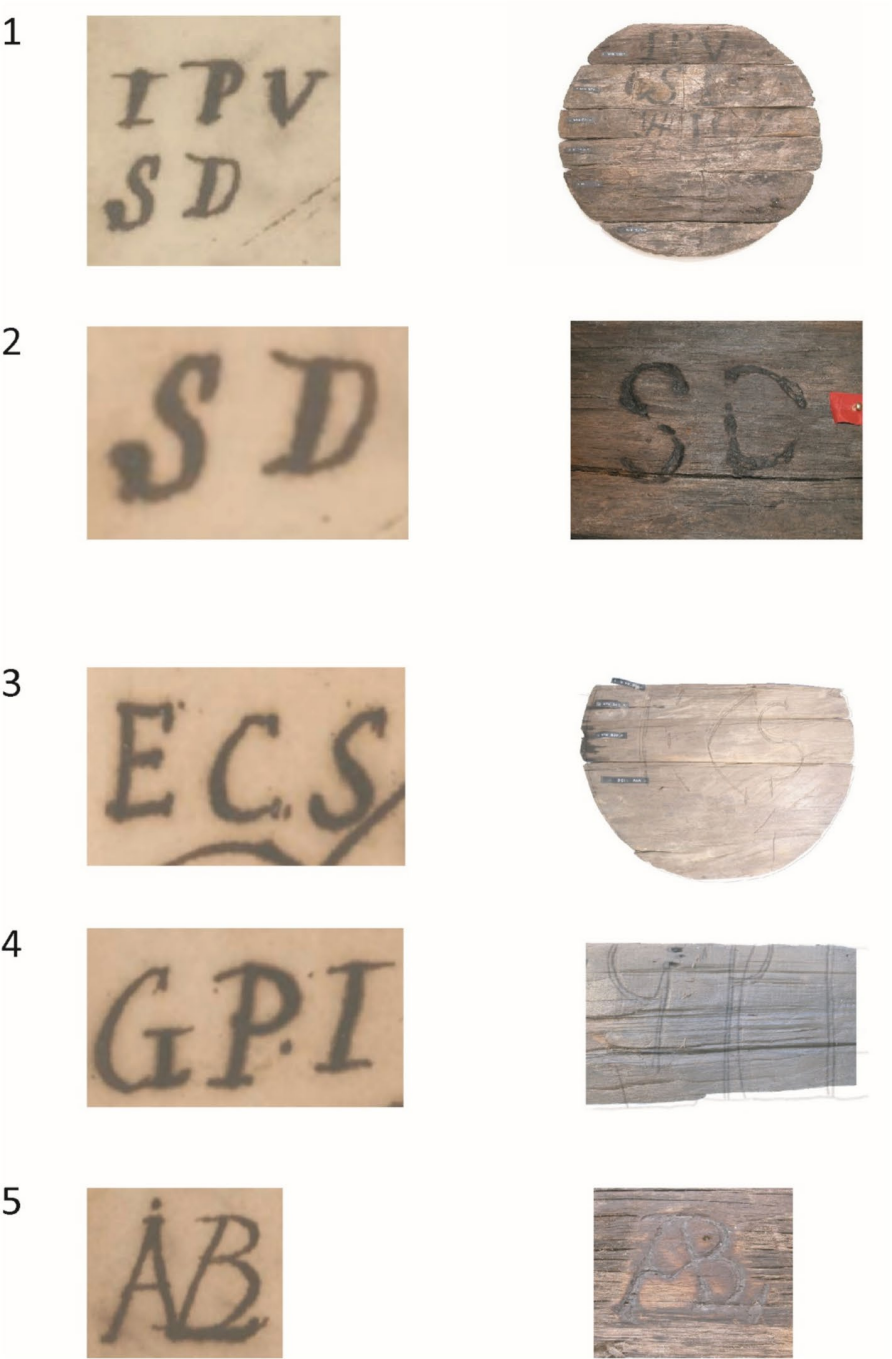
Cask marks found on BZN4 and*’t Hart*

**Fig. 10:**
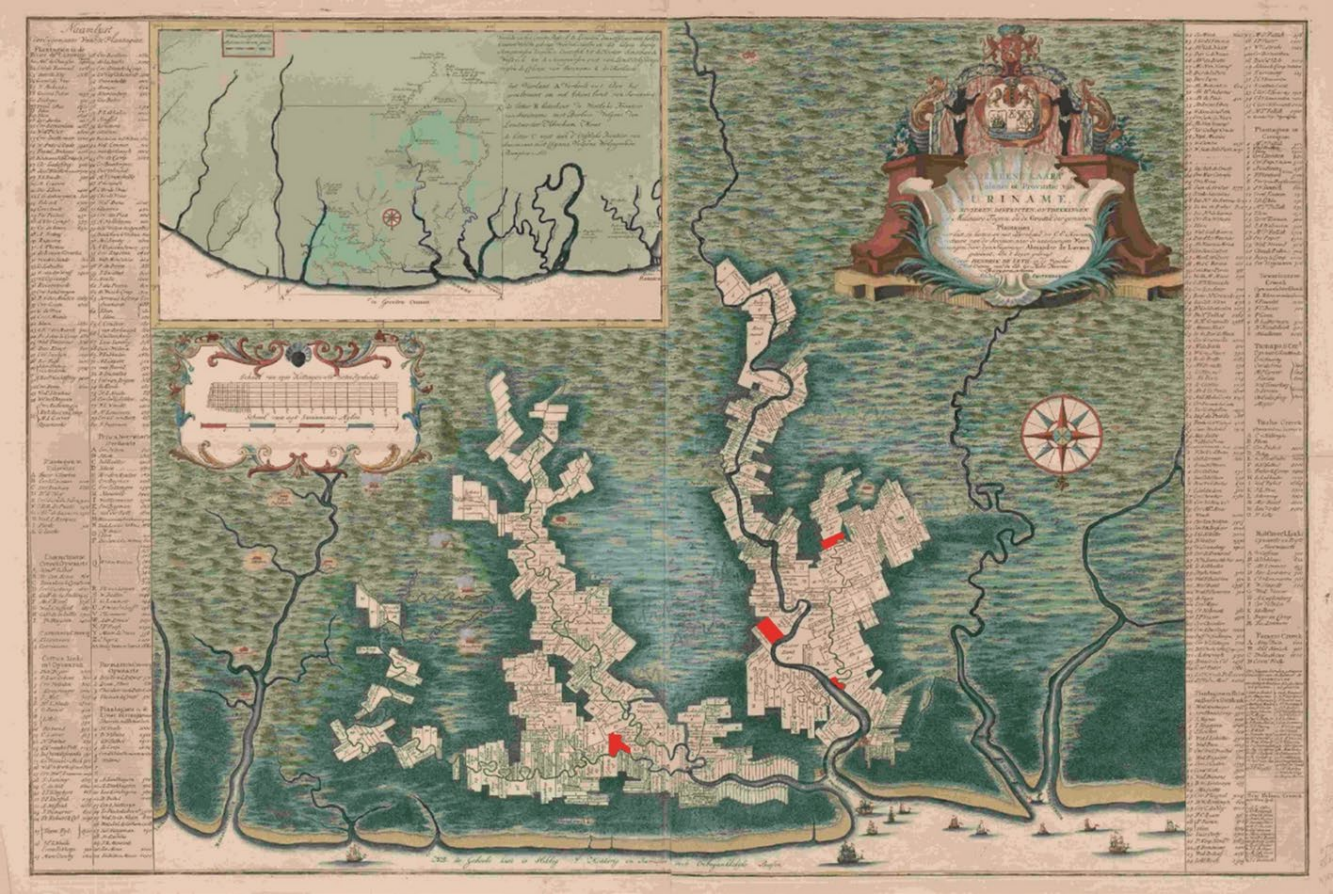
Map of the plantations in Suriname, 1737–1757. The plantations marked in red were owned by Jan Pietersz Visser. His mark, IPV SD, was on casks carried on *‘t Hart* and discovered on BZN4

Nevertheless, the marks found on BZN4 and*’t Hart* were compared, resulting in a partial overlap. Five marks were found on both BZN4 and the cargo of*’t Hart* on her final journey: IPV SD, SD, ECS, GPI, and ABL (Fig. 9). There were multiple versions of the subscript of the mark IPV (Jan Pietersz Visser): SD, OB, RR, and a three-leaf clover. Each subscript corresponds to one of Visser’s plantations (Fig. 10).^25^ When multiple variants of this mark were registered on the receipts of the same ship’s cargo on earlier voyages, they were registered together.^26^ Only IPV SD was present on*’t Hart* according to the cargo receipts. On BZN4, IPV SD is also the only known variant present. This mark indicates that the cask and its contents are connected to Jan Pietersz Visser and the plantation Sardam. The mark SD was also used separately on the BZN4 casks. In the casks marked with IPV SD or SD, coffee was found. However, on the receipt, these casks were associated with sugar. The marks ECS, GPI, and ABL also contained sugar according to the receipts. However, these casks were not sampled, making it impossible to compare the content.

Another noteworthy mark, MSP, was found on only one cask from BZN4 and was not recorded in the cargo receipts of *’t Hart*. The mark belonged to Matheus Sigismundus Pallak.^27^ Arent Hartjens,*’t Hart’s* husband, handled all of Pallak’s business affairs in Amsterdam, beginning in 1742. In 1746, Hartjens sold casks of coffee marked MSP.^28^ It is likely that the casks marked MSP did not require consignments because the ship’s captain, representing Hartjens, oversaw the delivery, hence they were not listed in the cargo receipts.

Various cooper’s tools were salvaged from BZN4. Mathijs Nieuwerkerk was the ship’s cooper. Looking for marks on the cooper’s tools for further confirmation yielded no results.^29^

## The Geographical Location of the Sinking

### BZN4

BZN4 was discovered on the Burgzand (Fig. 1), which served as part of the roadstead of Texel from the 16th to the 18th century. The shipwreck lies at a depth ranging from approximately 7.5 to 11.0 m, with an orientation nearly perfectly aligned north–south (Vos 2012, 167).

### *’t Hart*

Several crewmembers made a statement about the foundering of*’t Hart*. These accounts serve as the basis for the following description of the ship’s sinking, with particular attention being paid to the location of the ship on the fateful day.^30^

On 1 September 1751,*’t Hart* set sail from Suriname, carrying a cargo of sugar, coffee, and cocoa, bound for Amsterdam. On 26 November, after a voyage of 87 days, the ship and its crew reached the coast of Holland. Visibility was poor due to dense fog and drizzle. Around 11:00 AM, the crew realized they had entered the Zuiderhaaks, a vast area of shallows and sandbanks west of the Marsdiep, the approach to the Texel roadstead. Somewhere in this shallow area, the ship struck a sandbank forcefully, losing its rudder. Pieces of the hull broke off and floated to the surface. The ship stuck fast, and the crew discovered that it had sustained leaks from the impact.

Several distress shots were fired to call for assistance from a pilot and rescue ships. The tide was apparently rising, as after about half an hour, the ship refloated. It then bounced over the shallows until it reached deeper waters. From there, the crew maneuvered it through the channel—the Marsdiep—toward the east side of Texel—the intended first stop at the roadstead. In the channel, a barge with a pilot arrived from Den Helder. Eight men from the barge came aboard*’t Hart* to assist with pumping, while the barge itself was used to help steer the rudderless ship through the channel toward the safety of the roadstead.

Around 1:00 PM,*’t Hart* passed through the channel and was immediately maneuvered toward the Zuidwal. This was a shallow coastal area located directly to the east of the northern tip of Holland, forming the southern boundary of the Texel roadstead. The goal was likely to bring the damaged ship into shallow waters, to prevent it from sinking. However, a strong wind from the south-southwest pushed the ship toward deeper waters. To prevent this, two anchors were deployed, one after the other, but both anchor lines broke, causing*’t Hart* to be blown in a northerly direction.

The crew then dropped a third anchor, which held, but by that time the ship had already been blown far from the Zuidwal and ended up in deeper water. After an hour, despite the efforts to pump out the water, the ship was slowly sinking. Additionally, the strong wind had shifted from south-southwest to south. They no longer thought it would be possible to bring the ship back to the shallow Zuidwal, but hoped to bring it to the shallow Texel shore, across the deep water. To attempt this, the line of the third anchor was cut, and they tried to steer the ship in the desired direction by setting the jib, hoping the wind would propel the ship towards Texel. Unfortunately, they were unsuccessful.

The ship sank deeper and deeper, listing to one side, which was initially counteracted by the barge from Den Helder, which had been brought alongside. Then the sinking increased rapidly, and there was no time to save any cargo or personal belongings. Eighteen of the 25 crew members were transferred to the barge, and around four or half past four in the afternoon (16:00–16:30), the ship disappeared beneath the water. Left behind were former captain Pieter Orelius, the master, the cook, the sailmaker, two sailors, and an unknown passenger. The survivors were brought ashore in Den Helder at around six to seven o’clock in the evening (18:00–19:00).

### Comparative Analysis

The exact location of the sinking of a ship is rarely mentioned in textual sources, and the statements regarding the sinking of*’t Hart* are no exception. However, a rough indication of the location, specifically between the Zuidwal and the east coast of Texel, can be established from the statements.

The location where*’t Hart* began to sink does not necessarily match the location where the shipwreck eventually landed on the seafloor. A strong south-southwest wind could have blown the sinking ship in a north north-easterly direction. Additionally, the direction of the tide would likely have influenced the precise location where the ship ended on the seabed.

BZN4 was found approximately at the longitude where*’t Hart* had sunk, but it is located further to the north than its last-mentioned position off the Zuidwal (Fig. 1). Given the unknown influence of the currents and tide immediately after the sinking, the location where BZN4 was found provides no reason to dismiss the identification of BZN4 as*’t Hart*.

## Date of Shipwreck

### BZN4

The sinking of BZN4 was dated in the early third quarter of the eighteenth century. This is based on the collective dating of the pottery assemblage, with an upper limit around the third quarter of the eighteenth century (Vos 2012, 183). This corresponds to the estimated construction date between 1740 and 1750, based on the dendrochronological dating of the ship’s construction and the maximum life expectancy of a merchantman, which is typically between 15 and 25 years (Vos 2019, 96–97, 112–119, 120–121).

### *’t Hart*

*’t Hart s*ank in the roadstead of Texel on 26 November 1751.^31^

### Comparative Analysis

The dating of BZN4 early in the third quarter of the eighteenth century corresponds with the date of*’t Hart*’s sinking on 26 November 1751. However, the archaeological data were reexamined to determine whether a more specific date for the sinking of BZN4 could be established and to compare it with the sinking date of*’t Hart*, focusing primarily on the cask marks and pottery.

The mark IPV SD found on casks in BZN4 indicates a sinking date between 1747 and 1756. As mentioned earlier, IPV stands for Jan Pietersz Visser, who was married to Anna Dufour, and SD stands for the plantation Sardam. However, Visser only began marking his casks with his initials at the beginning of 1747. Prior to that, the casks were marked with FLW after Frans Lorens Wriedt, the original owner of Sardam and Anna Dufour’s first husband, who passed away in 1745.^32^ In March 1756, Anna married for the third time, at which time the mark was changed to BVW, the initials of her third husband, Lodewijk, Baron van Wangenheim. This makes it most likely that BZN4 sank between 1747 and 1756.^33^

We also reassessed the ceramic assemblage found on BZN4. During the archaeological investigation, it was noted that due to the limited number of ceramics and their poor datability, the sinking date could only be estimated with a wide margin of error. The reassessment of the pottery and the cask marks show that the sinking date of*’t Hart* in 1751 could align with the BZN4 finds. Therefore, this date supports the positive identification of BZN4 as the frigate*’t Hart*.

## Conclusion

The main objective of this paper has been to assess whether it is possible to identify BZN4 as the frigate*’t Hart*, with an initial outline of the details of BZN4 relevant to the identification. The next step involved a point-by-point comparison of the archaeological data from BZN4 with the information from textual sources about*’t Hart*, ultimately determining that*’t Hart* is a very strong candidate for the identification of BZN4.

### Specifications of BZN4 for Identification

The details of BZN4 that were important for its identification include the construction date, dimensions of the ship and its use as a merchantman. A crucial element of BZN4, however, is the cargo, specifically the cask marks. The use of these marks provides a direct connection to historical records of*’t Hart*. Additionally, the location and sinking date were also examined, further establishing a link with*’t Hart*.

### Justification for Identifying BZN4 as*’t Hart*

The identification of BZN4 as the two-deck frigate*’t Hart* is strongly supported by a convergence of archaeological evidence and historical records. First of all, the construction date of BZN4 was compared to the known construction date of*’t Hart.* Dendrochronological analysis of BZN4’s timber indicates a construction period around 1740–1750, which, after reassessment of the dendrochronological dating, was adjusted to 1736–1746. This timeframe aligns perfectly with the known construction date of*’t Hart*, that is, 1739.

The archaeological research provided insight into the dimensions of BZN4, allowing for an estimate of the ship’s length to be between 35 and 37 m and a beam of 9 m, based on a 17th-century assumption. These estimates were also reassessed using the length-to-beam ratios of frigates built in the Dutch Republic in the first half of the eighteenth century. This resulted in a reconstructed length of 30.6 m and a beam of 9 m, which closely matches the documented length of*’t Hart* at 30.8 m and beam of 8.4 m.

One of the most compelling pieces of evidence supporting the identification of BZN4 as *‘t Hart* is the cargo. On BZN4 the cargo consisted primarily of marked casks filled with coffee beans, which is what*’t Hart* was carrying according to documented evidence. In total, five marks on casks found on BZN4 were also found in the cargo receipts of*’t Hart*, while only 47 of the estimated 780 casks on board BZN were documented during the excavation.

A comparison of the two ships was initiated based on the matching marks and this provided the opportunity to conduct extensive archival research on*’t Hart*. This revealed that Arent Hartjens, who acted as husband and likely named*’t Hart*, conducted business with Pallak, owner of the MSP mark found on BZN4. Deeds show that Hartjens managed Pallak’s business affairs in Amsterdam and was involved in the trade of coffee beans marked with Pallak’s initials. This business relationship provides a direct link between the shipwreck and*’t Hart*. This was also evidenced in the cargo found on BZN4, which consisted of coffee and some cocoa beans packed in casks, matching the registered cargo of*’t Hart*, which included coffee, cocoa, and sugar.

In summary, the identification of BZN4 as*’t Hart* is justified by the strong alignment of archaeological findings with historical textual sources. The convergence of construction dates, cargo type, cask marks, and business relationships between key figures, provides a robust foundation for this hypothesis. Each piece of evidence corroborates other evidence, forming a cohesive and convincing argument.

The identification of the shipwreck BZN4 as the two-deck frigate*’t Hart* offers a unique opportunity to delve into the maritime history of the Dutch Republic and its colonial trade in the eighteenth century. By combining archaeological findings with archival research, a detailed picture of the ship, its cargo, and its final voyage has been reconstructed. This multi-faceted approach not only strengthens the identification, but also highlights the importance of integrating archaeological and archival research to uncover and understand historical narratives.

## Archives

### Gemeentearchief Zaanstad, Zaandam (GAZ)

- Oud Notarieel Archief Zaandam (ONAZ) [OA-0020]
- Oud Rechterlijk Archief Westzaan [OA-0008]

### Nationaal Archief, Den Haag (NA)

- Doop-, Trouw- en Begraafboeken (DTB) van Suriname [1.05.11.16]
- Oud Notarieel Archief Suriname (ONAS) [1.05.11.14]
- Sociëteit van Suriname (SvS) [1.05.03]

### Noord-Hollands Archief, Haarlem

- Collectie kaarten Rijkswaterstaat vóór 1850 [269]

### Stadsarchief Amsterdam, Amsterdam (SAA)

- Archief van de Directie van de Oostersche Handel en Reederijen [78]
- Archief van de Schout en Schepenen (SS) [5061]
- Oud Notarieel Archief (ONA) [5075]

## Data Availability

The cask marks referenced in the article can be viewed in the online database for cask marks at https://www.woodancaskmarks.org/.

## Footnotes

1 For an overview of Dutch shipwrecks and their identification, see the webpage of the Cultural Heritage Agency of the Netherlands (RCE), MaSS: stepping stones of maritime history, URL: https://mass.cultureelerfgoed.nl.

2 Nationaal Archief, The Hague (NA), Oud Notarieel Archief Suriname (ONAS) no. 760/439, 15 August 1748; Stadsarchief Amsterdam (SAA), Oud Notarieel Archief (ONA) no. 10223/349, 25 June 1742; no. 9181/442, 13 July 1745; no. 12789/67, 3 March 1746. For information about Pallak, see https://suriname.nu/surinamezoeken/knowledge-base/pallak-matheus-sigismundus.

3 SAA, ONA no. 10716/653, 2 August 1745; ONA no. 9432/14406, 8 December 1751; ONA no. 9432/14413, 21 December 1751.

4 The deeds are available on the webpage of the SAA: https://archief.amsterdam.

5 Archief van de Sociëteit van Suriname is available on the webpage of the NA, https://www.nationaalarchief.nl/onderzoeken/archief/1.05.03.

6 NA, SvS no. 268/728 (OBP), 20 May 1740.

7 SAA, ONA no. 10716/653, 2 August 1745; ONA no. 9147/1003, 24 November 1739.

8 Gemeentearchief Zaanstad (GAZ), Oud Notarieel Archief Zaandam (ONAZ) no. 5942/101, 13 December 1749.

9 Cornelis Jacobsz Ouwejan built the frigate *Vrouw Margaretha* (106 × 29.5 × 13.5 × 5.5 ft), comparable to *‘t Hart*, which sailed to Suriname in 1753. Enthoven Dataset.

10 GAZ, Oud Rechterlijk Archief Westzaan no. 1755/1738 (scan 18/9); no. 1755/1739 (scan 82/3).

11 SAA, ONA no. 9751/27, 13 February 1731; ONA no. 10716/652, 2 August 1745; ONA no. 12449/622, 24 September 1779.

12 SAA, ONA no. 10716/653, 2 August 1745; ONA no. 9147/1003, 24 November 1739.

13 One Amsterdam *voet* (foot) = 0.283 m; one *duym* = 2.57 cm. https://mgw.meertens.knaw.nl/maat.

14 SAA, ONA no. 10716/653, 2 August 1745.

15 If we use the average dimensions of Ouwejan’s frigates from the 1740s, with an average 3.6:1 ratio from the Enthoven database, a beam of 9 m would suggest a ship length of 32.4 m, while a beam of 8.5 m would indicate a length of 30.6 m. As with most archaeological shipwrecks, exact dimensions remain uncertain, and only carefully reconstructed estimates can be made.

16 NA, SvS no. 200, 13 November 1745, 13 March 1747; SvS no. 201, 26 October 1749, 29 March 1751; Postma Suriname Dataset, available on the webpage of DANS, https://dans.knaw.nl.

17 NA, SvS no. 199, 22 July 1744; SAA, ONA no. 9419/13390, 17 April 1748; NA, SvS no. 202, 20 April 1751; SAA, ONA no. 9432/14406, 8 December 1751; ONA no. 9432/14413, 21 December 1751; SAA, Archief van de Schout en Schepenen (SS) no. 2843, 7 March 1752.

18 WVW-569, WVW-506, WVW-560, WVW-765-1, WVW-765-2, WVW-1092, WVW-1093, WVW-1456 and WVW-1806.

19 WVW-569, WVW-506, WVW-765-1, WVW-1092 and WVW-1456.

20 WVW-1093.

21 Written communication from Bert van der Lingen, 18 April 2024.

22 NA, SvS no. 202, 30 August 1751.

23 The volume was calculated using the formula: V = πr2h. The calculation is based on the length of 116 cm and a diameter of 60 cm for cask AA (Vos 2012, 175).

24 This calculation is based on the weight of 494.09 g per *Amsterdamse pond* (Amsterdam lb). See the webpage of the Meerens Instituut, https://mgw.meertens.knaw.nl/plaats/44.

25 NA, SvS no. 280/318 (OBP), 24 October 1747. SD = Sardam, OB = Ornamibo, RR = Rorac and the three-leaf clover for Claverblad. https://www.allesurinamers.org/databestanden-met-zoekfunctie.

26 NA, ONAS no. 760/201, 19 July 1748; ONAS no. 760/657, 13 February 1750.

27 NA, ONAS no. 760/439, 15 August 1748.

28 SAA, ONA no. 10223/349, 15 June 1742; no. 12789/67, 3 March 1746; no. 12789/123, 18 April 1746.

29 SAA, ONA no. 9432/14413, 21 December 1752; SAA, SS no. 2843, 7 March 1752.

30 SAA, ONA no. 9432/14406, 8 December 1751; ONA no. 9432/14413, 21 December 1751.

31 SAA, ONA no. 9432/14406, 8 December 1751.

32 NA, SvS no. 280/318 (OBP), 24 October 1747; SAA, ONA no. 9179/267, 24 & 26 April 1745; https://www.allesurinamers.org/databestanden-met-zoekfunctie.

33 NA, Suriname DTB no. 3/119, 19 March 1756; SAA, ONA no. 10789/1396, 4 November 1760.
